# Editorial: Antiviral drugs and vaccines against important human respiratory viruses

**DOI:** 10.3389/fphar.2022.1082652

**Published:** 2022-12-07

**Authors:** Minghui Yang, Yang Yang, Qingbing Zheng, Khrustalev Vladislav Victorovich, Chenguang Shen

**Affiliations:** ^1^ Key Laboratory of Molecular Medicine and Biotherapy, Key Laboratory of Medical Molecule Science and Pharmaceutics Engineering, Advanced Research Institute of Multidisciplinary Sciences, Beijing Institute of Technology, Beijing, China; ^2^ Shenzhen Key Laboratory of Pathogen and Immunity, National Clinical Research Center for Infectious Disease, State Key Discipline of Infectious Disease, Shenzhen Third People’s Hospital, Second Hospital Affiliated to Southern University of Science and Technology, Shenzhen, China; ^3^ State Key Laboratory of Molecular Vaccinology and Molecular Diagnostics, National Institute of Diagnostics and Vaccine Development in Infectious Diseases, School of Life Sciences, School of Public Health, Xiamen University, Xiamen, China; ^4^ Department of General Chemistry, Belarusian State Medical University, Minsk, Belarus; ^5^ BSL‐3 Laboratory (Guangdong), Guangdong Provincial Key Laboratory of Tropical Disease Research, School of Public Health, Southern Medical University, Guangzhou, China

**Keywords:** antiviral agent, human respiratory virus, antiviral mechanism, host-virus interactions, therapeutics to viral infection, molecular diagnostics

Important human respiratory viruses that cause respiratory infections (such as coronavirus, influenza virus, adenovirus, and respiratory syncytial virus) have been one of the most significant causes of morbidity and mortality worldwide, posing unprecedented threats to global public health, especially the ongoing COVID-19 pandemic. As editors of this Research Topic, it’s our pleasure to summarize the main findings and perspectives detailed within each of the accepted articles.

This Research Topic compiles seven articles, including two reviews and three original research articles and two clinical trials from scientists in the field. The content of each article is summarised below.

Liu et al. give an overview of the Toll-like receptors (TLRs) pathway that participates in the SARS-CoV-2 pathogenic processes and acts as a pattern recognition receptor (PRRs). As we all know, SARS-CoV-2 can invade multiple organs of the body through Angiotensin I converting enzyme 2 (ACE2), causing excessive immunity, and leading to serious organ failure and even death. The SARS-CoV-2 infection could activate many TLRs such as TLR2, TLR3, TLR4, TLR7/8 and TLR9, and induces corresponding downstream response pathways, to produce type I IFNs to limit SARS-CoV-2 infection, or to activate the release of proinflammatory cytokines which may result in excessive accumulation of proinflammatory cytokines. Therefore, some TLRs agonists or antagonists might be used in the different stages of SARS-CoV-2 infection. Subsequent studies of regulatory TLRs drugs should also pay attention to the dual role of TLRs in the progression of COVID-19 disease.

Chan from The University of Manchester, United Kingdom, has developed fusion reporter gene assays (FRGAs) as models for plasma membrane and alternative fusion pathways as well as syncytial fusion in the SARS-CoV-2 which showed more sensitive, adaptable and unbiased than morphological fusion assays. The specificity of FRGAs has been confirmed using neutralizing antibodies and specific protease inhibitors. They found that syncytia formation is enhanced by TMPRSS2 or trypsin using the FRGAs coupled with morphological fusion criteria. More importantly, FRGAs have important implications in the development of universal blockers and synergistic therapeutics in SARS-CoV-2 infection.

In terms of drug discovery, Li et al. evaluate the potential effects and mechanisms of quercetin for the treatment of patients with colon adenocarcinoma (COAD) and SARS-CoV-2 infection by using bioinformatic analysis. COAD-related transcriptome data and COVID-19-related transcriptome data were used to obtain COAD/COVID-19-related genes (CCRG). A total of 105 potential target genes containing FOS, NFKB1, JUNB and JUN were found which may be involved in the treatment of quercetin in COAD/COVID-19 patients. However, the clinical use of quercetin needs to be further explored.

Jiang et al. characterize the breadth and efficacy of an isolated human monoclonal antibody (mAb) MW3321, which is much more resistant to escape mutation compared with another clinical staged SARS-CoV-2 neutralizing mAb MW3311. mAb-MW3321 could effectively reduce viral burden in hACE2-transgenic mice challenged with either wild-type or Delta SARS-CoV-2 strains through viral neutralization and Fc-mediated effector functions. Moreover, MW3321 exhibits a typical hIgG1 pharmacokinetic and safety profile in cynomolgus monkeys. These data support the development of MW3321 as a monotherapy or cocktail against SARS-CoV-2-related diseases. As described above, Antibodies have attracted a lot of attention for the diagnosis and treatment of infectious diseases. Nanobodies are generally more heat stable, easier and less expensive for production, and more amenable to protein engineering compared to conventional antibodies. Mei et al. reviewed nanobodies’ construction methods and potential functions in human infectious diseases. Recently, several nanobody therapeutics have been approved by the US Food and Drug Administration for various immune diseases, which brings hope for applying nanobodies in respiratory infectious diseases, especially for COVID-19.

A Randomized Controlled Clinical Trial was conducted to find that combined Povidone-iodine 0.5% and Glycyrrhizic acid 2.5 mg/ml (PVI-GA) nasal and oropharyngeal spray accelerates both laboratory and clinical recovery of SARS-CoV-2 infected patients in the early phases of the disease and reduces the household spread of the virus. It is worth mentioning that patients in the treatment group recovered quickly from taste and smell sensations, which greatly alleviates the discomfort of COVID-19 patients. Moreover, there was a notable reduction in transmission of the virus among the household close contacts in the treatment group compared with placebo (4% vs. 76%). Another Randomized Controlled Clinical Trial was conducted by Zou et al. Molnupiravir was found to significantly accelerate the SARS-CoV-2 Omicron RNA clearance in patients with COVID-19. Of patients receiving molnupiravir, 18.42% achieved viral RNA clearance on day 5 of treatment, compared to the control group (0%) (*p* = 0.0092). On day 7, 40.79%, and 6.45% of patients in the molnupiravir and control groups, respectively, achieved viral RNA clearance (*p* = 0.0004). More importantly, molnupiravir has a good safety profile, and no serious adverse events were reported. This study has important implications for the control and treatment of SARS-CoV-2 Omicron strains currently circulating worldwide.

The editorial team is grateful to all the authors and review editors for their contributions to the special Research Topic. We all hope that the relevant research in our Research Topic could provide useful information for the treatment of the SARS-CoV-2 infection.

